# Disruption of Fas-Fas Ligand Signaling, Apoptosis, and Innate Immunity by Bacterial Pathogens

**DOI:** 10.1371/journal.ppat.1004252

**Published:** 2014-08-07

**Authors:** Adam J. Caulfield, Wyndham W. Lathem

**Affiliations:** Department of Microbiology-Immunology, Northwestern University Feinberg School of Medicine, Chicago, Illinois, United States of America; Duke University Medical Center, United States of America

## Fas-Fas Ligand Signaling Induces Host Cell Death by Apoptosis

Fas ligand (FasL, CD95L) is a type-II membrane protein within the tumor necrosis factor (TNF) superfamily of death receptors [1]. FasL shares 25%–30% sequence homology with related family member proteins such as tumor necrosis factor alpha (TNFα) and TNF-related apoptosis-inducing ligand (TRAIL), with the most similarity present in the C-terminal homology ectodomain that extends into the extracellular space for receptor binding [2]. FasL engages and trimerizes the death receptor Fas (CD95) on cell surfaces to initiate the extrinsic apoptosis pathway [3]. The Fas-FasL interaction recruits the Fas-associated death domain adapter protein (FADD) via death domain binding, which interacts with dimerized procaspase-8 to form the death-inducing signaling complex (DISC) [4]. Caspase-8 catalyzes its autoactivation, followed by the proteolytic conversion of downstream effector caspases such as caspase-3 and -7 into their mature forms [5]. Effector caspases direct cell death by apoptosis, which results in nuclear and cytoplasmic condensation followed by cellular fragmentation into membrane-bound apoptotic bodies [6]. Caspase-activated DNase (CAD) cleaves genomic DNA between nucleosomes to generate short fragments prior to cellular condensation and membrane blebbing [7]. Membrane fragments are usually taken up by other cells and degraded in phagosomes via a process known as efferocytosis. Efferocytosis of apoptotic cells contributes to the resolution of inflammation by rapidly clearing cytotoxic cellular debris, and defects in this process can lead to inflammatory diseases such as acute lung injury [8].

Although FasL is expressed by many cell types, it is primarily recognized as associated with activated T lymphocytes and natural killer (NK) cells. FasL-dependent apoptosis plays important roles in tissue remodeling and the deletion of potentially autoreactive thymocytes to maintain immune tolerance during development, and it also contributes to tumor cell clearance by effector NK cells [9]–[11]. Though apoptosis has traditionally been defined as noninflammatory during these processes, FasL-induced cell death has been shown to be highly proinflammatory in the context of microbial infections [12].

## Apoptosis Stimulates Inflammatory Host Defenses

In response to many bacterial pathogens, the host responds by triggering FasL-dependent cell death as an inflammatory innate immune response [13]. Fas-mediated apoptosis of epithelial cells induces the release of proinflammatory cytokines, including TNFα, interleukin 8 (IL-8), macrophage inflammatory protein 2 (MIP-2), monocyte chemotactic protein 1 (MCP-1), and interleukin-1 beta (IL-1β) [14], [15]. In addition to these cytokines, Fas signaling positively affects CXC chemokine production that leads to enhanced neutrophil infiltration [16]. This apoptotic response is usually a protective mechanism by the host during bacterial infections. Optimal levels of cell death may eliminate replicative niches for intracellular pathogens and enhance further immune cell recruitment through the secretion of cytokines and chemokines, while excessive cell death often leads to an exaggerated immune response, self-tissue damage, and possibly death of the host. Experimentally, the contribution of FasL to inflammatory diseases can be assessed using C57BL/6 *FasL^gld^* mice, which contain a single residue mutation (F275L) within FasL that prevents binding to the Fas receptor [17]. Similarly, Fas-FasL signaling may be studied using *Fas^lpr^* mice, which lack a functional Fas receptor and thus cannot be activated by FasL [18]. In models of pulmonary inflammation, these mice exhibit reduced airway epithelial cell apoptosis, cytokine secretion, neutrophil influx, and tissue damage [19]. Similar results are obtained during knockdown of Fas by small interfering RNA (siRNA) [20].

During pneumonias caused by infection with *Pseudomonas aeruginosa*, FasL has been identified as a central regulator of innate defenses and inflammation. The presence of *P. aeruginosa* in the lungs leads to FasL-dependent apoptosis of host cells; the experimental use of *FasL^gld^* mice with an intranasal *P. aeruginosa* infection model shows increased disease severity with more rapid sepsis as compared to wild-type controls [21]. Adoptive transfer of bone marrow cells between wild-type and *FasL^gld^* mice determined that the protection conferred by FasL-dependent apoptosis was due to FasL interactions among lung epithelial cells during infection [21]. Epithelial cell apoptosis may consequently result in the secretion of defensins and cytokines that promote a stronger immune response. A similar role for FasL in host defenses is seen during infection with the stomach pathogen *Helicobacter pylori*: infection leads to Fas-dependent apoptosis of gastric epithelial cells [22]. Consistent with the role of Fas-FasL in enhancing immunity to bacterial infections, *FasL^gld^* mice produce lower levels of interferon gamma (IFNγ) from splenocytes and have enhanced disease severity compared to wild-type mice [22].

**Figure 1:**
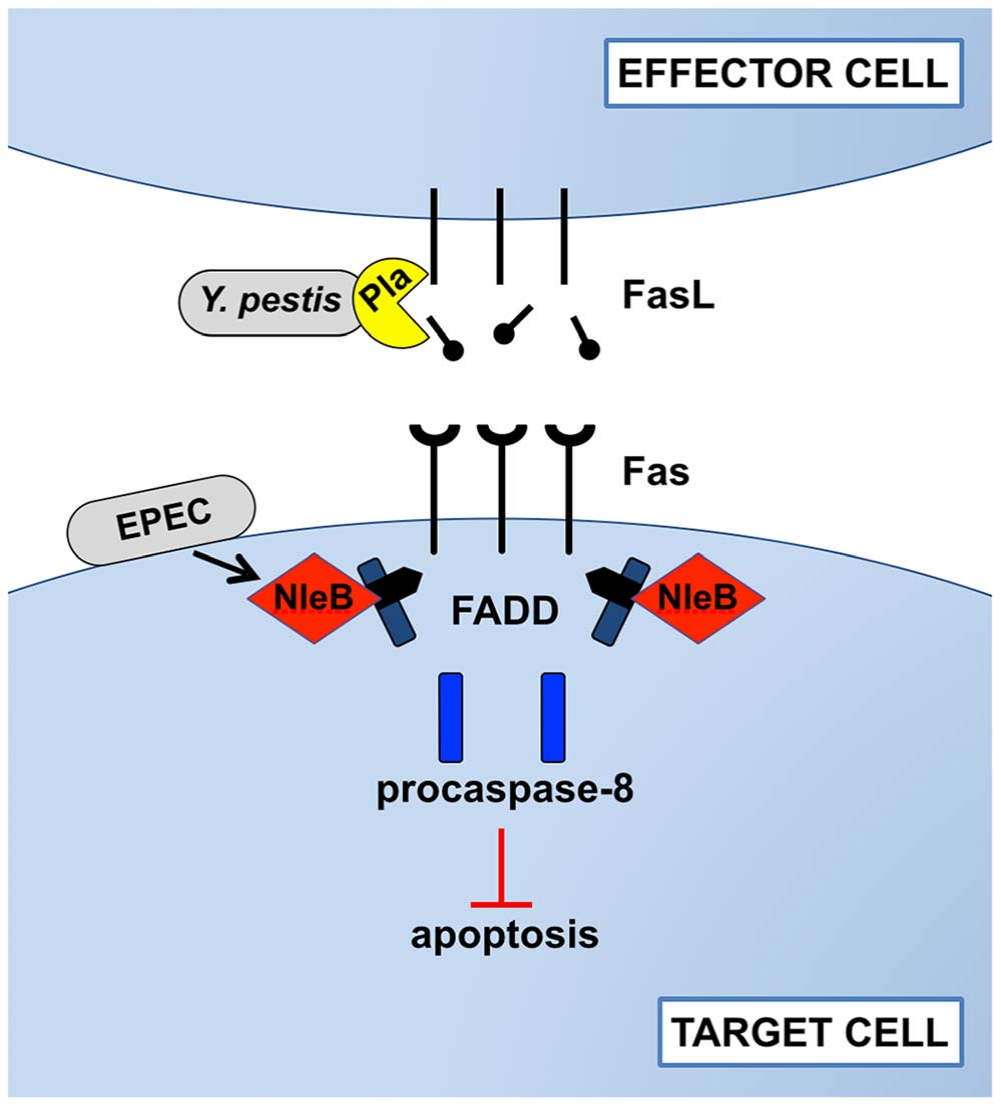
Disruption of Fas-FasL signaling by Pla of *Y. pestis* and NleB of EPEC. In response to bacterial infections, the host attempts to induce Fas/FasL-dependent cell apoptosis. During pneumonic plague, however, the Pla protease of *Y. pestis* directly cleaves FasL on effector cells to prevent the initiation of Fas signaling, blocking the activation of the initiator caspase-8, effector caspases -3 and -7, and cell death by apoptosis. As an alternative strategy during gastrointestinal infection, EPEC injects the type-III-secreted effector NleB into the cytoplasm of target cells, where it modifies FADD with N-acetylglucosamine to prevent death domain binding and downstream signaling following the engagement of Fas by FasL. While the mechanisms by which these bacteria target Fas-FasL signaling are distinct, the end result is the same: inhibition of apoptosis.

Some bacterial pathogens have developed virulence strategies to alter apoptosis during infection. For instance, following inhalation, macrophages phagocytose *Chlamydia pneumoniae* as a normal host defense mechanism. To evade killing of infected macrophages and to enhance pathogenesis, *C. pneumoniae* blocks cytochrome C release by the cell, thus inhibiting apoptosis via the intrinsic pathway [23]. Similarly, the virulence factors SidF of *Legionella pneumophila* and AvrA of *Salmonella* Typhimurium suppress apoptosis by inhibiting Bcl2 family proteins and by blocking c-Jun N-terminal kinase (JNK) signaling, respectively [24], [25]. Recently, several studies have described previously unknown mechanisms by which two bacterial pathogens overcome cell death–mediated host defenses by directly targeting the Fas-FasL signaling pathway. *Yersinia pestis*, the causative agent of the disease plague, prevents Fas induction by cleaving and inactivating FasL on the surface of effector cells, while enteropathogenic *Escherichia coli* (EPEC), a cause of gastrointestinal infections, post-translationally modifies FADD within target cells to arrest Fas-induced apoptosis (Figure 1).

## *Y. pestis* Prevents Apoptotic Cell Death by Cleaving FasL

We recently reported that *Y. pestis* prevents Fas-FasL signaling as a distinct pathogenic strategy to reduce apoptosis and enhance disease during mammalian infection [26]. *Y. pestis* produces the plasminogen activator Pla, a surface-exposed protease that is critical for the progression of the pneumonic (respiratory) form of plague and is required for bacterial outgrowth in the lungs [27]. While cell death via apoptosis predominates during the early, anti-inflammatory stage of pneumonic plague, as the infection progresses, cellular apoptosis is reduced as the synthesis of Pla increases and the bacterial burden rises [28], [29].

Our study showed that the Pla protease alters pulmonary apoptosis by directly cleaving both soluble and membrane-bound FasL to block the activation of Fas [26]. Through the extracellular degradation of FasL on the surface of effector cells, Pla prevents the induction of Fas-dependent caspase-3/7 activation in target cells, thereby actively manipulating host innate defense responses (Figure 1). In a murine model of primary pneumonic plague, the loss of active FasL in *FasL^gld^* mice reduces caspase-3 activation in recruited immune cells, including neutrophils, and allows for the enhanced outgrowth of *Y. pestis* Δ*pla* bacteria in the lungs. As a consequence of FasL cleavage by Pla, immune cell recruitment and inflammatory cytokine production are altered, while local tissue damage accumulates, resulting in increased pulmonary edema. In the absence of Pla, however, increased caspase-3/7 activation is observed in multiple cell types recruited to inflammatory lesions within the lungs, demonstrating a direct role for Pla in preventing host cell apoptosis during pneumonic plague. Specific inhibition of caspase-3/7 with the peptide inhibitor DEVD recapitulates the loss of FasL by enhancing bacterial outgrowth and reducing cytokine secretion, indicating that the cleavage of FasL by Pla overcomes these caspase-3/7-dependent host defenses. This work describes a previously unknown mechanism by which *Y. pestis* controls host cell death pathways and the activation of Fas signaling through the proteolytic degradation of FasL.

While a role for Pla in altering caspase activation was not previously known, the manipulation of host cell apoptosis by pathogenic *Yersinia* species via the Yop-Ysc type III secretion system (T3SS) is well established. The conserved *Yersinia* T3SS effector protein YopJ (also known as YopP in *Y. enterocolitica*) has been shown to enhance apoptosis in vitro under artificial secretion conditions [30]. During its evolutionary divergence from *Y. pseudotuberculosis*, however, *Y. pestis* acquired mutations within YopJ that reduce the efficiency of its translocation, which results in decreased caspase activation and diminished apoptotic activity [31]. This evolutionary loss of YopJ cytotoxicity led to the increased virulence of the plague bacillus [32], [33]. Additionally, neutrophils are resistant to YopJ-induced apoptosis, which is particularly relevant for plague pneumonia as neutrophils are the primary immune cell type recruited to the lungs [34]. Indeed, we showed that YopJ has no impact on caspase-3/7 activation during pneumonic plague, regardless of the presence or absence of Pla. Therefore, as opposed to the gastrointestinal *Yersiniae* that lack Pla and have a more cytotoxic variant of YopJ, the virulence of *Y. pestis* is enhanced by coordinate efforts to reduce apoptosis via the direct inactivation of FasL by Pla and the concomitant suppression of YopJ function, particularly in the lungs.

Over the past 1,500–20,000 years, *Y. pestis* has evolved away from a gastrointestinal lifestyle towards one that favors extraintestinal environments. Therefore, a shift in virulence strategy to prevent apoptosis during the later stages of disease may reflect an adaptation to the host response of the organs and tissues that the plague bacillus now infects. In the lungs, it is possible that cells injected and reprogrammed by the *Yersinia* T3SS may be recognized by the host and thus targeted for clearance via caspase-3/7-dependent mechanisms, with associated activation of the innate immune response. Indeed, injection of the T3SS effector YopK is known to stimulate apoptosis of pulmonary macrophages, and the inhibition of Fas-FasL signaling by Pla may therefore act to limit the recognition and clearance of these cells, resulting in an altered cytokine response in the lungs.

## Enteropathogenic *E. coli* Antagonizes Fas Signaling through Effector Secretion

As with pathogenic *Yersinia* species, enteric bacterial pathogens have also been shown to manipulate apoptotic signaling to successfully colonize the gut [35], [36]; however, direct interactions with the Fas-FasL signaling pathway were only discovered recently. Two independent groups showed that the EPEC T3SS effector NleB disrupts FADD-mediated apoptosis downstream of Fas-FasL engagement within target cells to counteract host defenses and enhance colonization [37], [38]. After injection into host cells, the N-acetylglucosamine (GlcNAc) transferase activity of NleB post-translationally modifies FADD at a single arginine residue (Figure 1). This residue is conserved among the related proteins TNF receptor type-1 associated death domain protein (TRADD) and receptor-interacting serine/threonine protein kinase 1 (RIPK1), which are also modified by NleB. GlcNAcylation of these proteins prevents death domain oligomerization and thus aborts apoptotic signaling downstream of the TNF family death receptors TNFR1, Fas, and TRAIL. The GlcNAc transferase activity of NleB is specifically required for bacterial gut colonization in a mouse model of EPEC, suggesting that EPEC and related pathogens disrupt Fas-induced apoptosis to overcome the otherwise protective host response conferred by this signaling pathway.

## Therapeutic Potential of Cell Death Modulators

It is becoming clear that the manipulation of cell death is a major strategy by which bacterial pathogens enhance virulence, although the specific mechanisms through which this occurs appear to be different from species to species. Some pathogens actively promote host apoptosis, while others inhibit Fas-FasL signaling. Understanding the in vivo effects of FasL on the virulence of a pathogen is made even more complex since different microbes stimulate varying levels of cell death (and by different pathways) and are likely to produce factors that both induce and abrogate apoptosis, with fine-tuning of cell death pathways for maximal virulence.

Despite our limited mechanistic understanding of cell death manipulation by pathogens, therapeutics that prevent bacterial virulence factors from modulating apoptotic cell death may have broad implications. Since both Pla of *Y. pestis* and NleB of EPEC disable Fas-FasL signaling to promote virulence, the administration of proapoptotic compounds, exogenous FasL protein, or agonistic antibodies that trimerize Fas may serve as possible therapeutics [39]. These treatments may help to overcome the manipulation of innate immunity by specific pathogens or to enhance the immune response more generally. Inhibitors to specific bacterial virulence factors such as Pla of *Y. pestis* or NleB of EPEC may also confer more targeted protection.

Preliminary studies have been conducted using the related apoptotic ligand TRAIL. When exogenous TRAIL is administered during pneumococcal pneumonia, apoptosis levels are restored, *Streptococcus pneumoniae* colonization is reduced, and mouse survival is enhanced, demonstrating the viability of increased apoptosis to bolster host defenses [40]. As the role of apoptosis in modulating host defenses becomes more clear, it will be important to validate the use of cell death manipulators as therapeutics for each infection model, since apoptosis may be detrimental to the host under certain circumstances, such as during systemic bacterial sepsis [41]. Continued investigation of the mechanisms by which pathogens manipulate apoptosis to alter host responses, via a combination of intracellular or extracellular activities on either effector cells or target cells, will provide insight for the development of future therapeutics.
